# Statistical assessment and visualization of synergies for large-scale sparse drug combination datasets

**DOI:** 10.1186/s12859-019-2642-7

**Published:** 2019-02-18

**Authors:** Arnaud Amzallag, Sridhar Ramaswamy, Cyril H. Benes

**Affiliations:** 1 The Center of Cancer Research, Massachusetts General Hospital, 149 13th Street, Charlestown, MA 02129 USA; 2 Harvard Medical School, Boston, MA USA; 3 Broad Institute of Harvard and MIT, Cambridge, MA USA; 4 Harvard Stem Cell Institute, Cambridge, MA USA; 5 Harvard-Ludwig Center for Cancer Research, Boston, MA USA; 6 Current Address: PatientsLikeMe, 160 Second Street, Cambridge, MA 02142 USA

**Keywords:** Drug combination, Synergy, High-throughput screen, Cancer therapy, Cancer cell lines, Melanoma, Statistical modeling, Visualization, Bliss independence

## Abstract

**Background:**

Drug combinations have the potential to improve efficacy while limiting toxicity. To robustly identify synergistic combinations, high-throughput screens using full dose-response surface are desirable but require an impractical number of data points. Screening of a sparse number of doses per drug allows to screen large numbers of drug pairs, but complicates statistical assessment of synergy. Furthermore, since the number of pairwise combinations grows with the square of the number of drugs, exploration of large screens necessitates advanced visualization tools.

**Results:**

We describe a statistical and visualization framework for the analysis of large-scale drug combination screens. We developed an approach suitable for datasets with large number of drugs pairs even if small number of data points are available per drug pair. We demonstrate our approach using a systematic screen of all possible pairs among 108 cancer drugs applied to melanoma cell lines. In this dataset only two dose-response data points per drug pair and two data points per single drug test were available. We used a Bliss-based linear model, effectively borrowing data from the drug pairs to obtain robust estimations of the singlet viabilities, consequently yielding better estimates of drug synergy. Our method improves data consistency across dosing thus likely reducing the number of false positives. The approach allows to compute *p* values accounting for standard errors of the modeled singlets and combination viabilities. We further develop a synergy specificity score that distinguishes specific synergies from those arising with promiscuous drugs. Finally, we developed a summarized interactive visualization in a web application, providing efficient access to any of the 439,000 data points in the combination matrix (http://www.cmtlab.org:3000/combo_app.html). The code of the analysis and the web application is available at https://github.com/arnaudmgh/synergy-screen.

**Conclusions:**

We show that statistical modeling of single drug response from drug combination data can help determine significance of synergy and antagonism in drug combination screens with few data point per drug pair. We provide a web application for the rapid exploration of large combinatorial drug screen. All codes are available to the community, as a resource for further analysis of published data and for analysis of other drug screens.

**Electronic supplementary material:**

The online version of this article (10.1186/s12859-019-2642-7) contains supplementary material, which is available to authorized users.

## Background

Drug combination can improve treatment efficacy and overcome drug resistance, combinations with synergistic effects are generally seen as superior to those with additive effect because they are more likely to provide efficacy not otherwise achievable with possibly added benefits of lower toxicities [1]. Large scale experimental testing of combinations is challenging however and predicting which combinations are synergic and in which context is difficult. In addition, for anti-cancer drugs it is clear that single drug efficacy varies widely depending on the genome of the targeted tumor [2, 3]. This suggests that context specificity will be possibly even more difficult to predict for drug combinations. Systematic drug combinations testing performed across large collection of cancer cell lines presents the opportunity to discover new beneficial drug combinations as well as to build better algorithms for the prediction of synergy. To identify synergism, several methods use surface dose response where all possible dose pairs within the chosen concentration ranges are tested (full dose matrix) [4, 5] and therefore requires a large number of different doses per combination: For instance, 9 doses per drug amounts to a surface of 81 data points. Hence, such methods are difficult to implement for testing a large number of drug combinations across many models. Experimental designs leveraging sparse drug dosage pairs allow for a tractable number of tests but the limited number of drug doses and/or the absence of replicates pose a challenge for the statistical assessment of synergism and antagonism.

We previously acquired a combinatorial drug data across 40 melanoma cell lines and 5778 combinations at 2 dose pairs. In this dataset, synergism was initially determined from a single combination data point (i.e. a single well) and two singlets (single drug wells). This large dataset did allow for the identification of novel combination with synergistic activity [6]. Nevertheless, experimental noise constitutes a challenge for broader data interpretation and further exploration of large-scale combination datasets. We thus aimed to develop a methodology to improve synergy calling and assign statistical value to synergies. In particular, we aimed to solve the issue of noise in the single agent data propagating through the synergy calculation: If the single agent effect of a given drug is incorrectly captured experimentally then all synergies calculated based on this estimate would be incorrect.

Several models to assess synergy have been proposed. Models can be separated into two distinct classes: effect based models, which for two drugs at a fixed dose, model the combination effect from single agent effects, and dose-effect based models, which model the dose response of the single agents and the combination (a dose response *surface* in the case of the combination - details of each method are reviewed in [7]). Briefly, the dose effect methods are mainly four methods: (i) combination subthresholding compares the combination effect with untreated cells using statistical tests. (ii) The highest single agent null model stipulates that the combination effect will be equivalent to the highest single agent effect. (iii) The additive effects postulate that non synergic combination is the sum of effects of the single agent; effect is defined as one minus viability. Finally, (iv) Bliss models the effect of a drug as a multiplicative factor applied to the number of cells tested compared to the untreated cells (i.e. the viability measure). Bliss independence stipulates that the combination viability is the product of the two singlet viabilities, as if the two drugs were applied successively. Note that this model holds whether the drug viabilities are less than one (killing cells) or greater than one (growing cells), and does not require the viabilities to be modelled as probabilities.

All these model have shortcomings, especially in the context of sparse dose response testing. Combination subthresholding requires replicates to perform the statistical tests. The Additive model does not have a clear physical model, and can lead to inconsistency, like predicting a negative number of cells when the sum of the two singlet effects is greater than 100%. The highest single agent model predicts the combination effect to be equal to the highest single agent effect. This prediction can be quite inaccurate in sparse dose testing, when there is only one dose per singlet and noise in the assay. The Bliss model can produce inflated viabilities when drugs are inactive, and therefore slightly greater than one by random chance. Here, we used a Bliss independence model based on logarithm transformation of the viabilities.

The main dose-effect model, Loewe additivity, requires determination of the singlet doses that achieve the same effect as the combination. In the dataset analyzed here, in 58% of the drug pair-cell line assays, one of the singlet does not reach the effect of the low dose combination. This prevented us to use Loewe additivity in this work. This is mainly due to the fact that a large range of doses was not tested in this screen: Loewe additivity is not suitable for analysis of sparse doses screens.

**Fig. 1:**
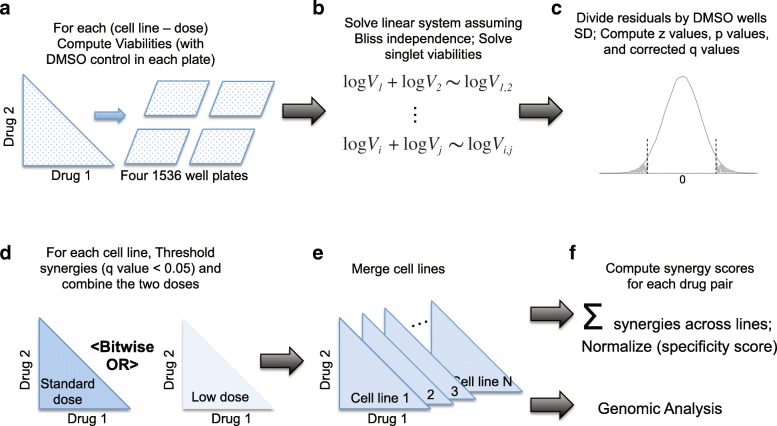
Analysis Pipeline. This figure describes the analytical pipeline, from the raw data to the synergy scores. **a** All pairwise combinations between the selected drugs (108) where platted in a pseudo-random order on four 1536 plates, and viabilities were computed by dividing the number of cells in the well by the mean number of cells in the untreated wells. **b** Combination viabilities were modeled with the Bliss independence assumption, after passing to the logarithm, yielding a linear model of 5778 equations modeling the combination viabilities, and 108 unknown, representing the singlet viabilities. **c** Residuals of the linear system were used as a score for synergy. Variance in the DMSO wells was used to model sample error on the measurement of combination viability, yielding *p* values and *q* values for each combination. **d** For each cell line, if one of the two dose showed synergy, well considered the combination synergic in that cell line. **e** We counted the number of cell lines were the combination synergy was significant (absolute synergy score). **f** We computed the synergy specificity score from the absolute synergy scores (see methods). Synergy scores could be modeled using genomic features, as they are available for most cell lines used here on the GDSC project website (www.cancerrxgene.org)

We reasoned that the combinations between a given drug A and the 107 other drugs in the screen contained recurrent and leverageable information about the singlet viability of drug A, and that this information could be used to overcome singlet data noise and overall experimental noise. Here, we use this concept of information redundancy built into the combination data to derive better estimates of the singlet viabilities. Based on this we further propose a method for the assessment of significance of synergy and antagonism as well as specificity of drug interaction. Our computational pipeline is graphically summarized in Fig. 1 and described in the Methods Section. We found that this method identified correctly drug pairs previously described as synergistic or expected to be synergistic based on previously published mechanistic studies. The method also captures a number of less expected synergies with good initial support in the literature. Below, we describe first the data and the shortcomings of the existing methods, especially the simple application of the Bliss model. Then, we describe this novel method and its advantages compared to the singlet versus combination viability derived Bliss score. We provide a concise and easily readable R code allowing users to reproduce our results even on a personal computer. In addition, we developed a web application that allows any user to quickly explore the results through an interactive drug-drug heat map.

## Results

### Data description and singlet noise propagation

**Fig. 2:**
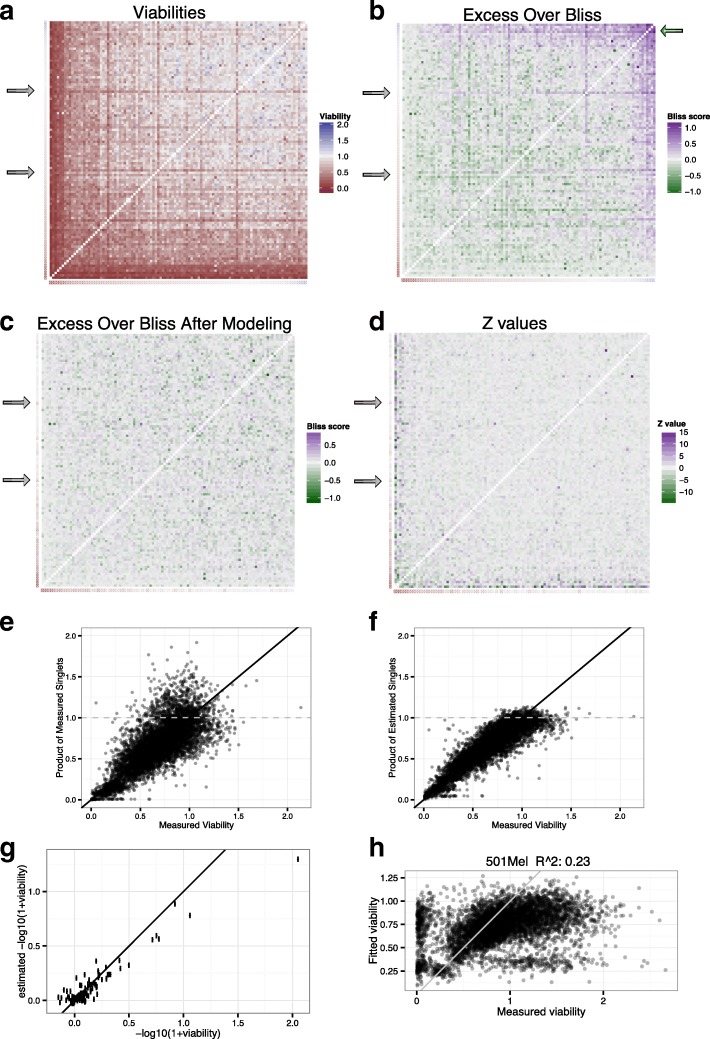
Heat maps of all pairwise drug combination results for cell line COLO792 low drug dose. **a** Viability (i.e. nuclei count divided by the average nuclei count in the DMSO treated wells) **b**-**c** Excess Over Bliss scores before (**b**) and after (**c**) linear modeling of the singlet viabilities (**d**) synergy Z values. In all heat maps rows and columns have the same order (sorted by singlet viabilities). Each heat map has a single row and single column of discs representing measured singlet viabilities (**a**-**b**) or estimated singlet viability by the Bliss linear model (**c**-**d**). Gray arrows indicate drugs where singlet viability was high compared with viabilities in combinations with other drugs, producing horizontal dark red rows in (**a**). This produces spuriously high Excess Over Bliss scores (**b**, gray arrows). Moreover, singlets with very high viability tend to produce a large number of high Excess Over Bliss scores even when the drug combination has no effect on the cells (**a**-**b**, top right corners). Such problems are not observed after the singlets are estimated from the linear model (c-d, top right corners and gray arrows) (**e**) Comparison between the solutions of the model (singlet viabilities) and the measured singlet viabilities (that were not used in the model). Error bars in the y axis indicate plus or minus 2 standard errors. Units of the model are shown: negative log_10_(1 + viability). **f** Model based on Bliss independence has a R squared of 0.90, indicating that it is a good model for the combination of drug effects. **g** Scatter plot of the singlet viabilities, experimentally measured versus estimated. The vertical error bars indicate the 95% confidence interval. **h** Scatter plot of combinations viabilities (measured versus estimated) for cell line 501MEL at high dose, the assay with the lowest R² in this dataset

In order to systematically explore a substantial number of drug combinations, we recently performed a large-scale drug screen across 40 melanoma cell lines using a limited number of drug doses. The drug combination response surface was limited to two concentration pairs: both drugs at a “standard” dose estimated to inhibit fully the intended target(s) while avoiding overly broad off target, or both drugs at low dose (1/5 of the standard dose) in order to further emphasize on target combination and synergy detection based on partial target inhibition [6]. Using this design, all possible pairwise combinations between 108 drugs were tested systematically (108*107/2 = 5778 pairwise combinations) on 40 cell lines, representing more than 439,000 data points. A heat map representing the 5778 combination viabilities for one assay (cell line COLO792 at high drug dose) is shown in Fig. 2a, where the intersection of row *i* and column *j* shows the viability of the combination of drugs *i* and *j*, and singlet viability are represented as crossed rectangles on the sides of the heat map. Viability is used to measure drug effect, and is defined as the number of cells in the treated well divided by the average number of cells in untreated wells.

Methods to determine drug combinations synergism encompass several mathematical approaches that each have advantages and drawbacks and while in many cases they can yield concordant conclusions that is not always the case [8]. In addition, in some cases a given method can also yield discrepant conclusions regarding whether the tested drugs have a synergistic effect or not [7]. The most commonly used methods can be broadly sorted in two types depending on which reference model they use: Those based on the Bliss independence hypothesis and those based on the Loewe Additivity principle [9, 10]. Large screening campaigns come with limit to the number of drug doses that can be tested to allow for high-throughput and manageable costs. In these conditions, Loewe Additivity based methods are not applicable because full dose response curve of each single agents (and preferably more than one combination of doses) is required to obtain a synergy estimate [7]. On the other hand Bliss hypothesis based models can be applied on sparse data as long as the singlet doses are also used in combination. The advantages and drawbacks these models have been discussed in details previously [8].

The Bliss model of independence states that if two drugs have independent effects, then the viability of the combination should be equal to the product of the viability of the two single drugs:

1 $$ {V}_i\bullet {V}_j={V}_{ij}. $$

Departure from this assumption will lead to a non-zero Bliss score defined as the difference between the left hand and the right-hand term of eq. (1):

2 $$ {S}_{ij}={V}_i\bullet {V}_j-{V}_{ij}. $$

Here, a positive Bliss score indicates that the observed combination viability *V*_*ij*_ is lower than expected when the drug effects are independent (the product *V*_*i*_ ∙ *V*_*j*_), therefore it indicates synergy. Conversely, a negative Bliss score indicates antagonism. While the Bliss score allows to easily rank synergies across tested combinations, the direct calculation of the Bliss score in an assay with no or few replicates poses several problems that we illustrate here using a large but sparse (dose-wise) combination dataset of drug pairs across a set of melanoma cell lines [6]:

(i) The single drug viabilities are used in many different combinations, and the error of measurement of single viabilities propagates to many Bliss scores because the error in measuring *V*_*x*_ propagates to the combination Bliss scores *S*_*xj*_ (for all 1 < *j* < *N*); for instance, when a singlet viability is overestimated, this will lead to overestimated Bliss scores for all combinations involving that singlet (Fig. 2b, gray arrows show purple stripes of likely overestimated Bliss scores in our test dataset). Indeed, at the same positions in Fig. 2a, we can see stripes of low viabilities for these two drugs, but the viabilities of the singlets are near 1, as indicated by the color of the discs on the left side of the heat map. One possible interpretation is that these two drugs produced synergies with almost all the other 107 drugs we tested; the implicit assumption made by simply calculating a Bliss score from this data (Fig. 2b). A more parsimonious explanation for the high number of synergies for combinations involving these drugs is that the viability of the singlet, measured only once in a single well of the 1536 well plate, was overestimated due to experimental noise. Therefore, the stripe of synergies indicated by gray arrows in Fig. 2b are likely false positives with very large Bliss values, and risk occulting true positives.
(ii) When single drugs have little or no effect, observed singlet viabilities are often greater than one (over 100% viability) due to random error on measurement, and their product can produce high Bliss scores even if the combination’s viability is greater than one. For instance, if two drugs have each a singlet viability of 1.2, and the combination viability is 1, the combination will have a large Bliss score of 1.2^2^ − 1 = 0.34 despite the fact that no substantial growth inhibition was seen with any of the 3 treatments. This problem is particularly visible in the top right corner of Fig. 2b (green arrow): when the singlets have high viabilities, the Bliss scores seem to be systematically high even though the combination had little effect (Fig. 2a top right corner); even more problematic for the interpretation of the full dataset, it seems that such Bliss score are among the highest in the full dataset as can be seen in the heat map. A simple approach is to cap all viability data at 1 but this is an ad-hoc solution that is inferior to approaches that could estimate the experimental noise and suppress it in statistically rooted manner.
(iii) if the experiment is noisy, any model will have a poor fit to the data: since the Bliss score is the deviation from the model of independence of drug effects, a noisy experiment will tend to yield over estimated Bliss scores overall: Since viability cannot be negative the data distribution is likely to be skewed towards positive synergy values (underestimating antagonistic interactions). Figure 2h shows an experiment with the poorest fit at high dose; it is also the cell line that gave the highest number of combinations with Bliss scores greater than 0.3.

To address these issues, we developed a drug combination data modeling approach that corrects for experimental noise of the single agent data, that is further overall robust to experimental noise and that provides robust estimates of synergy and associated *p* values. The model uses the full combination dataset to infer the activity of single drugs and identify synergies and associated *p* values. To illustrate our approach, we show the raw data for single agent activity and corrected (modeled) data in various graphical forms in Fig. 2 (a-g), focusing on the cell lines tested (COLO792) at the high concentration pair dosage.

Before applying our model, to determine the validity of applying the Bliss independence model, we compared the viability of each drug combinations with the product of the viability of the two single drugs (Fig. 2e). The Bliss model postulates that these two variables are equal if there is no synergy or antagonism. Inspection of the viabilities by scatter plot for each cell line shows that the two variables are related, as the combinations lie along the x = y line. Furthermore, there is approximately the same number of points in the upper left semi-plane and in the lower right, suggesting that there is not a strong bias towards synergy (upper left) or antagonism (lower right). Therefore, solving the model should give a reasonable approximation of the singlet viabilities, and for determining synergism from the data.

### Linear modeling of singlet viabilities with the Bliss model

To determine the singlet values from the combination dataset we linearized the Bliss equation (eq. 1) and applied a Bliss-based linear model to estimate the singlets with combination data only. We postulated a linear model where the singlet viabilities at a given dose are the unknowns (108), and used the 5778 measured combination viabilities to solve the singlet viabilities with far more accuracy than if we used the measured singlet viabilities. In Fig. 2f, we plotted the observed viabilities for cell line COLO792, against the product of the estimated viabilities by the linear model, as opposed to the product of the observed singlet viabilities plotted in Fig. 2e. The model fits the data very well (R^2^ = 0.90) and values are aligned along the x = y axis (black line). An interesting outcome of the singlet modeling is that the number of high predicted viabilities (viabilities greater than one) is greatly reduced (Fig. 2e, f, dots above the dashed horizontal line). Viabilities are not expected to be often greater than one, because cancer drugs are expected to kill or inhibit proliferation of cancer cell lines rather than improve proliferation over vehicle treated controls. This suggests that such high viabilities were largely due to noise in measurement of the singlets. As expected, high measured singlet viabilities (> 1) yielded overall high Bliss scores (Fig. 2b, high values in the top right corner). However, this trend was not seen when using the model-estimated singlets (Fig. 2c, d). Furthermore, singlet modeling suppressed outlier combinations where one singlet viability seemed to be overestimated and led to a stripe of overestimated Bliss score associated to one drug (gray arrows). These observations illustrated here on cell line COLO792 hold true for almost all the cell lines in the combination dataset analyzed (see below for statistics).

### Bliss models R^2^ show good fit and may be used for quality assessment

**Fig. 3:**
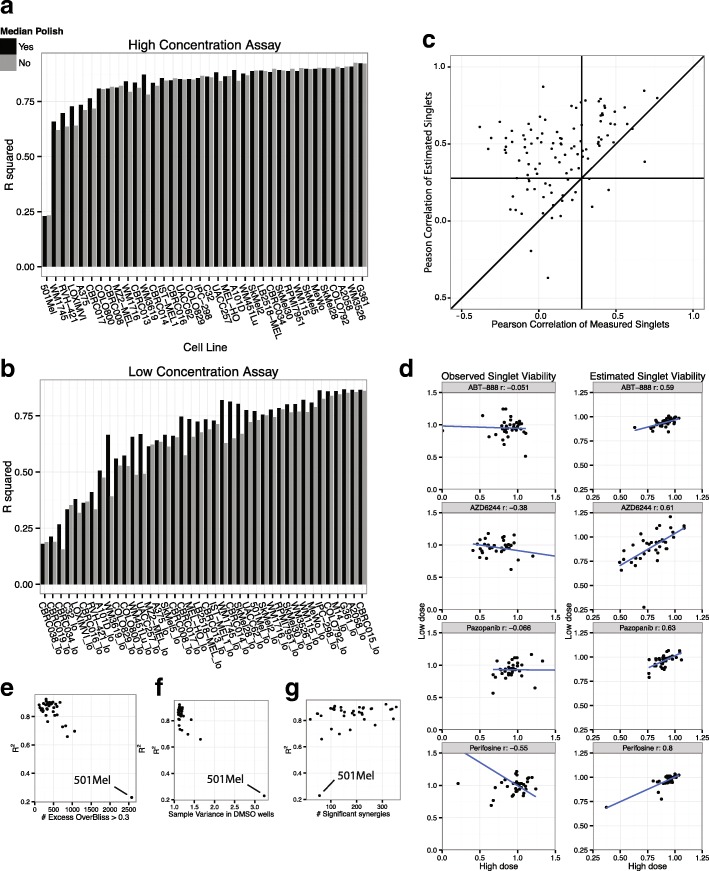
Linear modeling with bliss independence. **a**-**b** R^2^ values show the goodness of fit of the model for the high (**a**) and low (**b**) dose assays. Median R^2^ is 0.81, showing that the model fits well the majority of the data. Gray bars show R^2^ values when no median polish is performed in the pre-processing of the cell line plates, showing that median polish increases R^2^ values and probably reduce noise. **c** Pearson Correlation between low dose and high dose singlet viabilities across cell lines. Correlations are much higher when using the solved singlet viabilities than when using the viabilities measured on the plate. **d** Scatter plots of singlet viabilities between high and low dose, for measured singlets (left panels) and solved singlets (right panels). In these four examples, correlations went from negative to positive and significant. **e**-**g** R^2^ vs other measures on models at standard drug dose. **e** Negative correlation between the model R^2^’s and the number of synergy found using an arbitrary cutoff (> 0.3) on Excess Over Bliss (showing high dose assays only). The cell line with the worst R2 also had the most synergic combinations (more than 2500 out of 5778), most of them are probably false positives. **f** The number of significant synergies does not correlate with the models R^2^. **g** The sample variance measured from the DMSO wells is a surrogate for the experimental noise. It correlates with low R^2^ for models with R^2^ < 0.8; it suggests that very low R^2^ are mainly due to noise on measurement rather than an abundance of synergism or antagonism

After applying the Bliss linear model to all the cell line in the dataset, we found that the model fit the combination data very well across cell lines (median *R*^*2*^ of 0.81, Fig. 3a, b). In addition, we fitted models before and after median polish of the 1536 well plates: the median polish improved R^2^ values in most cases. Importantly, combinations were randomly assigned to the wells: neither the rows nor the columns of the 1536 well plates correspond to repeatedly the same drug. Therefore, there is no obvious reason for the median polish to increase R^2^ values, other than by removing experimental noise (Fig. 3a, b). Additionally, we compared the solved singlet viabilities to the measured singlet viabilities (throughout all singlet viability were left out of the linear modeling) we found excellent agreement between calculated and measured singlet values (median Pearson correlation of 0.82; Fig. 2g). We also computed the 95% confidence intervals around the solutions of the estimated singlets. Interestingly, for most cell lines, the confidence intervals are much smaller than the difference between the estimated singlet viability and the observed singlet viability from the screen (Fig. 2g, vertical bars), suggesting that we get more precise estimates of singlets viabilities from the linear system using the drug combinations than by using the observed viabilities in the single drug assay wells.

In the high dose assay, all cell lines had an R^2^ of 0.66 or above except 501Mel (R^2^ = 0.23, Fig. 3a and Fig. 2h). Interestingly, when not using singlet modeling, this is also the cell line with the most synergies (excess over Bliss score greater than 0.3): it shows 2577 synergies out of 5778 possible drug combinations in the high concentration assay (Fig. 3e), a proportion unlikely to be accurate. Indeed, we observed a high variance in observed synergy values, both in terms of R^2^ of our linear bliss models and in terms of viability in the DMSO (untreated) wells (Fig. 3f) suggesting high experimental noise for this particular set of plates: this high number of synergies was not observed at the lower dose assay. Therefore, it is overall unlikely that 501Mel is particularly prone to exhibit synergic drug interactions, and we concluded that this cell line data has a higher measurement error than the rest of the dataset. Because our method uses the variance in DMSO wells as the variance for the null hypothesis (no synergy or antagonism) it requires very strong deviation from the null hypothesis for significance in a case like 501Mel, reducing the impact of experimental noise onto the synergy calling. Indeed, our method detected a low number of synergies for 501 Mel, among the lowest compared to other cell lines (Fig. 3g). Therefore, by accounting for the variation in DMSO wells, our method automatically discounts many potential false positives, compared to the application of the Bliss formula directly to the raw data, and does not necessitates manual exclusion of this cell line from the data analysis.

### Application of the linear model increases consistency between the two drug doses

After we applied the singlet estimation for each assay (one cell line at one dose), we explored the consistency of the singlets across the cell lines between the high dose and the low dose pairs (Fig. 3c, d). Although the viability obviously depends on dosing, we expect some level of correlation between the two doses when considering all data available. We found that the correlations increased significantly when using the estimated singlets compared to the measured singlets (0.48 median correlation with estimated singlets, 0.14 with measured singlets, t test *p* value < 7*10^− 18^). Only 28 of the 108 observed singlets were significantly correlated between the high and the low dose, versus 79 estimated singlets (testing that the Pearson’s correlation is different from zero, with R function cor.test, *p* value cutoff of 0.05), rejecting the null hypothesis that the singlet viabilities are un-correlated between the two doses, for most of the 108 drugs. Further supporting the value of the approach, several drugs with a negative correlation between doses effect using the observed viabilities had a significant positive correlation of the estimated singlets (Fig. 3d). In addition to singlet viabilities, synergy values of drug combinations showed higher correlations across cell lines between the high and the low dose when using the Z value from the linear model than when using standard excess over bliss (*p* value < 2*10^− 28^, t test).

### Promiscuous drugs and specific synergies

**Fig. 4:**
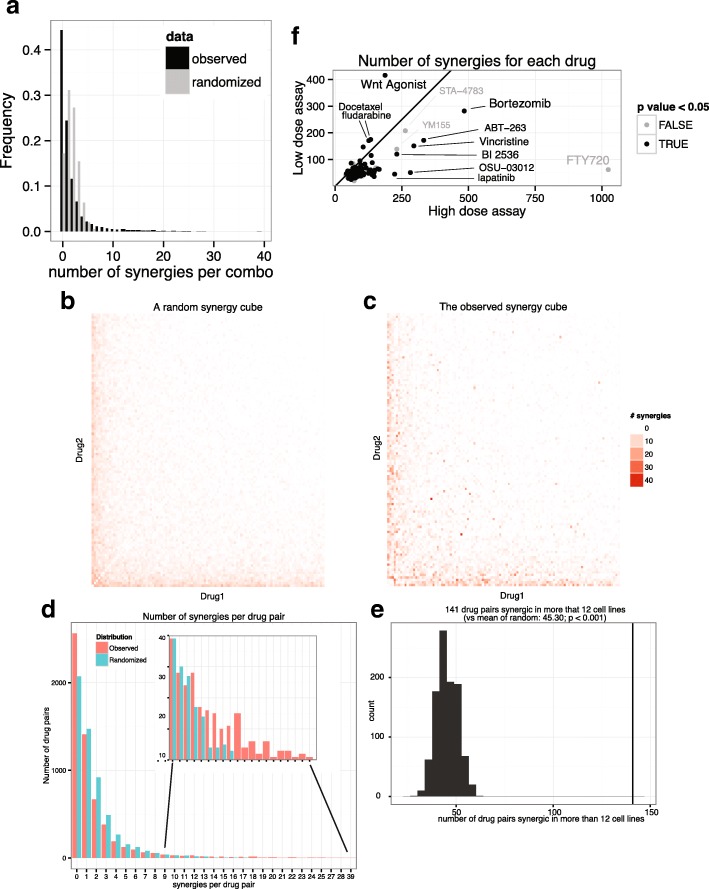
Synergy Square and histograms. **a** Black bars: Distribution of absolute synergy scores (i.e. the number of cell lines that showed synergy) for each tested drug pair. Gray bars: a randomization that conserved the number of synergies per cell line, but reassigned synergic drug pairs within a cell line. The observed distribution has much more 0 scores and high scores than the randomized one. **b**-**e** Randomization that conserves the sensitizing property of each drug. **b** A random absolute synergy score matrix that conserves the total synergy score per drug, in order to conserve each drug sensitizing properties in the randomization (generated with binomials distributions). **c** The observed absolute synergy score matrix. Drugs are ordered according to their sensitizing potential. **d** Comparison of the random and observed distributions (**e**) Number of synergy scores greater than 12, observed (vertical line) versus 1000 randomizations (histogram bars). **f** Sensitizing potential of each individual drug. For each drug, the number of synergies observed across all second drugs and cell lines, in the high dose assay versus the low dose assay. The number of synergies in common between the low dose and high dose assays was tested for each drug with the fisher test (black dots represent the 77 drugs with significant overlap)

For each drug, we then counted the number of synergies observed in each of its combinations (with 107 other drugs at either dose in 40 cell lines). We found a large range of synergy count among the 108 drugs, from 76 to 1065. Here also, we found good concordance between the assays at low drug dose and high drug dose: There was a positive correlation between the sensitizing potentials at both doses. For most of the drugs, high synergies occurrence at low dose is associated with high occurrence at high dose (Fig. 4f). We then asked for each drug if the specific synergies matched between the high and the low drug dose. We found a significant overlap of synergies between the two doses for 77 drugs out of 108 (71%, fisher test *p* < 0.05, black dots in Fig. 4f). Note that such synergies found at two doses increase our confidence in the results, since synergy was observed in two independent assays; nevertheless, observing a synergy at only one dose does not dismiss the observation as spurious, since the synergy profile of each drug may differ between the high dose and the low dose for a variety of biochemical reasons (for example the low doses might be insufficiently inhibiting targets to yield effect or the high doses might have single agent effects too pronounced to allow for synergy observation in a cellular viability assay).

Figure 4f shows that some drugs were engaged in many more synergies than others. Some of these broadly synergistic drugs that promote activity of many other drugs have been referred to as “promiscuous” [11]. Most of the promiscuous drugs showed a significant agreement between the high dose and the low dose, with the exception of FTY720/fingolimod, which had the highest number of synergies at high drug dose but not at low dose. FTY720 also had the lowest median singlet viability of all drugs at high dose (0.08). It is possible that the variance of the log ratio $\log \left(\frac{V_i\ast {V}_j}{V_{ij}}\right)$ may be underestimated when *V*_*ij*_ is very small, and therefore synergies involving FTY720 singlets should be considered with caution. We note however that FTY720 is also implicated in a large number of synergies when applying the excess over bliss formula to the raw data.

**Fig. 5:**
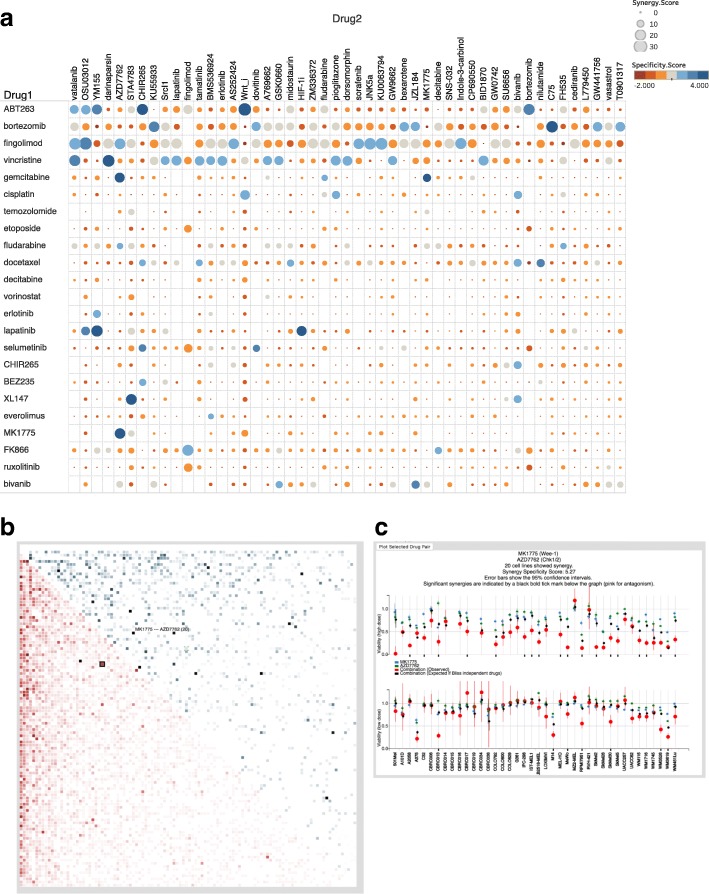
Snapshot of the Web Application and selected synergies. **a** Selected examples of synergies across some of the drugs: Absolute synergy (size of the dot) and specificity score (color code) are displayed. The top promiscuous drugs have a diverse profile of synergistic pairs and synergy pattern are different across different a selection of drugs synergies is. **b** The synergy score for all the drug pairs are displayed in a square heatmap. The lower triangle displays the absolute number of cell lines that displayed synergy for the drug pair, in a white to red color scale; the upper triangle displays the specificity score. When a drug pair is clicked, the corresponding viabilities for all cell lines is displayed in a dot plot. **c** Dot plot with the details on the synergy score. At the top the names of the drugs are shown, together with the absolute synergy score and the specificity score; then the detailed standard concentration results, and below the low concentration results, per cell line. The singlet viabilities for each drug estimated from the linear model are displayed in blue and green with standard error as a bar. The black dots show theoretical viability under assumption of independence of drug effect (no synergy). The red dots show the observed viability, with error bars as the standard deviation of the un-drugged wells. The error bars of the estimated singlet and the estimated combination under the independence assumption are the standard errors derived from the linear model (see methods). Hence, the distance between the black and red dots show the magnitude of the synergy (or antagonism). Significant synergies (p adjusted < 0.05) are shown with a black tick, and significant antagonism are shown with a pink tick

Most of the other drugs with a high number of synergies showed significant agreement between the high and low doses (Fig. 4f). Interestingly, ABT-263 (navitoclax) was seen as a top sensitizing drug. This is consistent with its targeting of anti-apoptotic proteins of the BCL2 family that is expected to lower the apoptotic threshold across cell lines and potentiate the pro-apoptotic effect of other drugs. We also found that the proteasome inhibitor bortezomib was a strong sensitizer, possibly due to the strong impact that proteasome inhibition has on many cellular processes. Several cytotoxic drugs (fludarabine, vincristine and docetaxel) are also among the top sensitizers. This might again be due to broad activity of these drugs across most cell lines inducing strong cellular stress and making cells more susceptible to many other additional stresses. Importantly, even with these broadly synergistic drugs, the pattern of synergy across partner drug (drug 2) was distinct indicating some level of specificity in sensitization. For example, there is little overlap among top synergy drug partners for the most sensitizing drugs (Fingolimod, Bortezomib, ABT-263, vincristine) (Fig. 5a, Additional file 1: Table S1). In addition, we identify lapatinib as a sensitizer. In contrast to the other sensitizing drugs, this is a much more specific inhibitor that targets receptor tyrosine kinases, primarily of the EGFR family. However, we recently demonstrated that several synergistic events identified for combination including lapatinib are actually due to its capacity to inhibit multidrug resistance (drug pumps that expelled a range of compounds from the intracellular space). In fact, this activity can be a major confounding factor in analyzing synergies with this and likely other “specific” drugs [6].

The sensitizing potential of a drug has been historically called potentiation; it has been described as a distinct effect from synergy, not specific to the biology of the drug combined, for instance when drugs act on pumps that expels other drugs out of the cell, leading to broad potentiation of the effect of these drugs [10]. Here, we distinguished specific synergy from promiscuous synergy and broad potentiation through the use of the specificity score (see Methods).

The specificity score compares the absolute synergy score of drug combination A-B with the mean of all the combinations including drug A and the mean of all the combinations including drug B. It allows to prioritize specific synergies over more general ones. Based on the specificity and the absolute synergies scores, we can establish a ranked list of drug combinations corresponding to strong drug interactions and highest confidence (Additional file 1: Table S1). Many of the top combinations have synergistic interactions supported by published studies and known biological functions. For example, AZD-7762 an inhibitor of the DNA damage response kinases CHK1/2 presents with high synergy scores with the Wee-1 inhibitor MK-1775 and high specificity score (Synergy score 20 and specificity score 5.2; top 0.1% with the top 1% score across 5778 combinations tested corresponding to a specificity score of 2.26). Wee-1 and CHK1/2 are known to regulate mitotic entry and progression and indeed combined inhibition was recently shown to be synergistic via forced mitotic entry [12]. We also identify the combination of AZD-7762 and gemcitabine (a DNA damaging agent) as the second top synergy among combinations with the CHK1/2 inhibitor as well as the combination with fludarabine another DNA damaging agent as the top 4th; These are expected outcomes for combinations of DNA damaging agents with an inhibitor of the DNA damage response. Addressing a different cancer pathway, the catalytic mTOR/PI3K inhibitor BEZ-235 was seen to display strong synergy with the non-catalytic mTOR inhibitor rapamycin – an outcome that is strongly mechanistically supported in a recent publication showing that rapalogues and catalytic mTORC inhibitors can suppress mTOR activity synergistically [13]. Among less expected outcomes we also flagged the combination between ABT–263 and Wnt agonist as highly synergistic with 28 cell lines showing synergy and a specificity score of 3.82 (top 5% overall). Although we are not aware of any literature evidence for this exact drug combination, an interesting corroboration is found in a large pharmacogenomics study, revealing that activating mutations of beta-catenin predict for sensitivity to ABT-263 [14]. The Wnt agonist in our screen would indeed be expected to recapitulate the effect of the beta-catenin mutation. Another set of synergies of interest are seen with stibogluconate, a compound with an unclear mechanism of action but that was recently shown to inhibit the tyrosine phosphatases SHP1 and SHP2. Our analysis identifies the MEK1/2 (MAPK Kinase) inhibitor selumetinib and the SRC inhibitors SU6656 and dasatinib as top synergies for stibogluconate. SHP2 is indeed a known positive regulator of the MEK1/2-ERK1/2 pathway [15] at least in part through activation of SRC [16] and other evidence supports a SRC regulatory role of SHP2 [17].

### A web portal for the exploration of synergies across large datasets

Our dataset of drug-drug synergies across melanoma cell lines can be represented by a cube of 108 drugs × 108 drugs × 40 cell lines. In order to allow rapid, in-depth exploration of this large dataset by other investigators, we designed an interactive, user-responsive, web application based on the d3 library [18]. The web-based portal (http://www.cmtlab.org:3000/combo_app.html) displays an interactive visualization of the drug by drug data square, symmetric with respect to each drug listed in identical order along the x and y axis (Fig. 5a). The color coding of the square represents the count of significant synergies obtained with the corresponding x + y combination across the 40 cell lines, or the synergy specificity score, or both, based on user preferences. With a mouse over or a click, the name of the combined drugs, the number of synergic cell lines and the synergy specificity scores are displayed above the data matrix, along with the known drug target, which allows easy look up of the drug names and targets with a search engine. In addition, to explore the third dimension of the cube (synergies behavior for a given combination across cell lines), a dynamic query is sent to the server when a data point in the matrix is selected, and the combinatorial viability data across all cell lines is instantly displayed as a dot plot (Fig. 5b). The dot plot shows viability of the drug combination (red) and the expected viability under the assumption of Bliss independence (black), with error bars. Significant synergies and indicated with a black tick near the cell line name, and significant antagonisms with a pink tick.

## Discussion

Combining drugs is largely considered a requisite for durable clinical responses in oncology, particularly in solid tumors. Rational combination building with small number of combinations testing remains very challenging and is unlikely to address the vast majority of clinical cases in the near future. Thus, systematic combination screening is needed in order to discover combinatorial therapeutic strategies. Furthermore, because of the known heterogeneity of tumors even within a given cancer type or genetically defined subtype (such as BRAF V600E melanoma) there is a need to perform these combination screens across large numbers of models. This would allow to understand how broadly effective across patients a candidate combination might be and ideally give insight into associated predictive biomarkers to be used for patient selection. Even for targeted therapeutics, toxicity is a major challenge and often limits efficacy because dose reduction becomes necessary during the course of treatment. Although, there are example of combinations being better tolerated than single agents (BRAF plus MEK inhibitors in melanoma) this is unlikely to be observed in the vast majority of cases. Hence, combination treatments are generally more toxic than single agent ones. Thus, it is largely considered that synergistic drug pairs (or higher order combinations) are preferable over those presenting with additive effect not only to yield efficacy not observed with single agents but also to minimize toxicity. Combinatorial drug screening is however technically and analytically challenging and very resource intensive. Thus, in order to screen large sets of tumor derived models, often compromises have to be made to allow for enough testing of each combination in a sufficient number of models. This translate generally into a reduction in the number of experimental tests aimed used to estimate each combination effect and synergy. Estimating synergy is complex in part because a given drug pair might not be synergistic in some dosing conditions but strongly so in others. For example, if the targets of the drugs are insufficiently engaged it is likely that this might not disturb their function sufficiently to observe effect or synergy. Unfortunately, for many drugs in development there is often insufficient information available to robustly determine which doses should be tested. Thus, sparse combination datasets represent an even greater challenge than more dense ones not only because of lack of technical redundancy but because of the potential to miss the conditions that would yield maximum synergy in the tests performed. Overall, these challenges call for a more robust estimation of synergy ideally allowing for a statistical estimate rather than relying on arbitrary choice of arbitrary threshold to designate outcome as synergistic. Here, we describe a method for the assessment of significance of drug combination synergies from viability cell line screens with sparse data that reduces noise in synergy calling and provides a statistical value for synergism for each test performed. To overcome the absence of replicates and low number of tested drug doses, we exploited information redundancy: Each drug was part of a large number of combination tests. To do so we used a Bliss based linear model of 5778 equations (the total number of pairwise combinations that were tested in the dataset) to yield better estimates of singlet drug viabilities and therefore yield better synergy estimates. To our knowledge, this is the first time the Bliss model is applied as a large linear model to solve for singlet viability with higher accuracy before computing excess-over Bliss scores. Because there is currently no other publicly available combination screening with sufficient overlap in drugs and cell lines with the one used here, we cannot rely on a gold standard set of results to measure the accuracy of our method, an issue that will be encountered by other researchers until a large compendium of combinatorial drug screening data in cancer cell lines is available. To quantitatively estimate the improvement that our approach, we used viabilities measured at different doses with the same combination in the same cell line as a proxy for replication. We showed that, after applying our regression method to estimate the singlets, the correlation between high dose and low dose singlets improved. In addition to the singlet viabilities, we also observed that our synergy Z values are moderately more consistent between high and low dose. We note however that we should not expect consistency of synergy to be high, since the difference between the two doses is quite large (5x), and synergy between two drugs might be observed only at one of the two doses. Estimation of error rate through large number of experimental replicates is prohibitive at scale but could nevertheless be built in the future to establish better gold standard for estimating error rate and advantages of new modeling methods, such as the one presented here.

Our model was designed to address challenges of synergy estimates and noise in sparse combination datasets. However, our approach could be applied to denser datasets such as those allowing to build drug dose response surfaces. In this case our method could be applied by considering each dose test separately and solving for singlets in a large Bliss linear model. Another beneficial aspect of our method is that it gives a *p* value per unique test (here each well in an assay plate) in the screen. In these cases of denser datasets, our method could also be extended to yield a single p value for a full combination surface response.

## Conclusion

Our analysis of a large combination dataset across cancer cell lines shows that the resulting matrix of data can be interpreted using the Bliss model of synergy. Based on the Bliss hypothesis, using linearization of the matrix of combination viability outcome, we efficiently and robustly identify statistically significant synergistic events directly from the combination data without relying on single agent data. Importantly, we present evidence that our approach reduces the noise in the dataset and allows for identification of context specific synergies. Unlike the traditional use of synergy calculation using the Bliss hypothesis directly on the primary experimental data or other approaches based on different models of synergy, our approach associates a statistical value to each combination outcome, allowing for a less biased decision of hit calling threshold. Taken together, by comparing our results with previously described synergies, we show that by deriving a robust synergy score across the full dataset we identify well defined combinations as well as more novel ones with initial supporting mechanistic evidence in the literature. In addition to a statistical (*p* value) score for each combination tested in each cell line we present an approach to determine a specificity score for each drug pair allowing for prioritization of hits for follow-up analyses and experimentation.

## Methods

### Overview of the analytics pipeline (Fig. 1)

The main statistical procedure to determine synergism is performed on a single cell line and a single concentration pair. The 5778 combinations are spread on four 1536 well plates (Fig. 1a). The transformation of the Bliss independence eq. (1) with the logarithmic function yields a linear system that can be solved to obtain single drug viabilities (Fig. 1b). Conveniently, the residuals of the model represent the deviation from Bliss independence, our null hypothesis, and therefore indicate synergy and antagonism. We tested whether the residuals (each residual corresponds to one drug combination) are significantly different from zero. The mean of the null distribution is zero, and the variance, ideally, should be the variance that is due to the error on measurement and other variability *not* due to interaction between the two drugs tested in this combination. We approximated the variance using the sample variance of the multiple un-drugged wells present in each plate (as a measure of the experimental noise) plus the standard errors of the solved singlet viabilities. Using this null distribution, we could assign *p* values to the residuals (Fig. 1c) that is the probability of a viability to be lower (resp. higher) than observed under the null hypothesis; This p value was used to determine synergism (resp. antagonism). The variance of the null hypothesis allows to attribute statistical significance to synergism in each cell line independently, and therefore serves as a normalizing factor to compare synergism scores of a given combination across various cell lines. The p value was corrected for false discovery with the Benjamini Hochberg method [19] over the 5778 drug combinations. We consider a drug pair to yield synergy in a given cell line if one of the two drug doses passed significance (Fig. 1d).

### Absolute score and specificity score

For each drug pair (*i*, *j*), we counted among the 40 cell lines how many showed synergy, and we called it the absolute synergy score *T*_*ij*_. (Fig. 5b, lower triangle; darker color shade corresponds to higher synergy score). Since some drugs are promiscuous and produce many more synergies that other drugs, we aimed at obtaining value compensating for this imbalance across the dataset. We computed “specificity score” for synergy events (Fig. 5b, upper triangle). The specificity score allows to better identify (i) cases where synergy is observed solely because of a promiscuous drug, from (ii) cases where the synergy score for the combination was above and beyond an expected absolute synergy score. The score compares the synergy (*i*, *j*), to all (*i*, *k*) and (*k*, *j*) synergies with *k* representing all other drugs tested in combination with i and *j*. Specificity score *Sc*_*ij*_ compares *T*_*ij*_ *to* all the synergies involving either drug *i* or drug *j* as follows:

3 $$ S{c}_{ij}=\min \left(\frac{T_{ij}-<{T}_{ik}{>}_{k\in D}}{SD{\left({T}_{ik}\right)}_{k\in D}},\frac{\ {T}_{ij}-<{T}_{kj}{>}_{k\in D}}{SD{\left({T}_{kj}\right)}_{k\in D}}\right) $$

Where <*T*_*ik*_>_*k* ∈ *D*_ is the average absolute synergy score of the combinations of drug *i* with all other drugs, and *SD*(*T*_*ik*_)_*k* ∈ *D*_ is the standard deviation. Therefore, the specificity score measures synergy effect that is above the synergy caused any potential promiscuous effect of drug *i* or drug *j*.

### Preprocessing and computation of viabilities

We median polished [20] the logarithm of nuclei counts using the rows and columns of the 1536 well plate (one iteration), and re-exponentiated the results. Using the logarithm for median polish avoids the occurrence of negative counts and negative viabilities. For each plate, we computed a *DMSO control value*: the trimmed mean on the control wells nuclei counts (10% trimmed on each side). Finally, we computed viabilities by dividing the nuclei counts by the plate’s DMSO control value*.* The median polish procedure increased the R^2^ of the independence model for almost all the cell lines tested, suggesting that it did remove noise in the assay (Fig. 3a-b).

### Significance assessment of synergy

Given or limited dose response coverage, we used Bliss independence to model synergy [9, 10] given by the Bliss score when ***S***_***ij***_ is null (no synergy):

$$ {\boldsymbol{S}}_{\boldsymbol{i}\boldsymbol{j}}={\boldsymbol{V}}_{\boldsymbol{i}}.{\boldsymbol{V}}_{\boldsymbol{j}}-{\boldsymbol{V}}_{\boldsymbol{i}\boldsymbol{j}}, $$

where ***V***_***i***_ is the viability of the singlet *i* and ***V***_***ij***_ is the viability of the combination of the drugs *i* and *j*. Since replicates are not available in our dataset, it is difficult to assess statistical significance of synergy after computing a Bliss score. Furthermore, single measurements of single drugs (singlets) are noisy and measurement error in one singlet propagates to all Bliss values that involve that singlet across *all* 108 combinations involving drug A, leading to inflated Bliss scores. In order to overcome these difficulties, we built a linear model based on the assumption that deviations from Bliss independence are centered on zero (i.e. synergism is as (in)frequent as antagonism). We reasoned that if this assumption holds the model ***V***_***ij***_~***V***_***i***_. ***V***_***j***_ should fit the data. Indeed, we observed that for the large majority of the cell lines the combination viability is similar to the product of the viability of the single agents, thus confirming the validity of the Bliss independence assumption for the drugs and doses we used (Fig. 2e).

### Solving the single drug viabilities from the combination viabilities

Since the Bliss model seems to fit the data, we used the log transform of the Bliss independence assumption [9, 10]. in order to create a linear model where a linear combination of the log singlet viabilities yield the drug combination viability:

4 $$ \left\{\begin{array}{c}{\boldsymbol{W}}_{\mathbf{1}}+{\boldsymbol{W}}_{\mathbf{2}}\sim {\boldsymbol{W}}_{\mathbf{1},\mathbf{2}}+\boldsymbol{\epsilon} \\ {}\dots \\ {}{\boldsymbol{W}}_{\boldsymbol{i}}+{\boldsymbol{W}}_{\boldsymbol{j}}\sim {\boldsymbol{W}}_{\boldsymbol{i}\boldsymbol{j}}+\boldsymbol{\epsilon} \end{array}\right. $$

where ***W***_***ij***_ ***=  −*** **log**_**10**_***V***_***ij***_, ***V***_***ij***_ is the viability of the combination of the drugs ***i*** and ***j***, ***W***_***i***_ ***=  −*** **log**_**10**_***V***_***i***_ corresponds to the single drug ***i*,** and ***ϵ*** is a random noise term centered on zero.

We solve the system of 5778 equations to obtain estimates for the 108 singlets at once. This avoids relying on measurement of one well for singlet viability determination: this system is largely over-defined as we use all the combinations to infer only 108 singlet viabilities. This allows an accurate estimates of singlets, and by extension, better estimates of synergy than relying on experimental singlet values (see results). In contrast, the Excess Over Bliss score (or Bliss score) is defined by: ***S***_***ij***_ ***= V***_***i***_***.V***_***j***_ ***− V***_***ij***_. The measurement error in one singlet ***V***_***i***_ propagates to Bliss scores ***S***_***ik***_ for all ***k*** within the 108 drugs.

### Number of synergies per drug pair and randomization of the synergy cube

The distribution of the number of synergies per combination found across cell lines was compared with randomizations of the synergy cube of two kinds. In the first method, we computed an indicator variable that indicates whether the synergy is significant, at a threshold of 0.05 FDR-corrected *p* value. We then randomly permuted the 5778 synergies within each cell line. We show that the observed distribution, compared to the randomization, 1) has more combinations that show synergy across a large number of cell lines and 2) has far more combinations that consistently show no synergy at all across the cell lines (Fig. 4a). Some drugs showed many synergies, but most did not (Fig. 4). The goal of the second randomization was to ask whether the non-random structure of the observed synergy cube was only due to the presence of promiscuous drugs that sensitizing the cells to many other drugs, or whether it was evidence of a number of specific synergies. We simulated the drug-drug matrix containing the number of synergies from a binomial distribution such that the number of synergies *per drug* is conserved in the random matrix. The binomial parameter *p* for combination *(i,j)* is function of the sum of two drug specific parameters *p*_*i*_ and *p*_*j*_; and N is 40 (for the 40 cell lines). 1000 random matrices were generated and the distributions compared to the observed distribution. None of the random matrices had large numbers of synergies as seen in the observed one (comparison of the number combinations with more than 12 synergies, *p* < 0.001; 141 drug pairs produced synergies in more than 12 cell lines compared to a mean of 45.30 in the randomizations – Fig. 4d-e).

### Web application for interactive data visualization

In order to allow rapid, in-depth exploration of this and other similar large datasets, we designed an interactive, user-responsive, web application based on the d3 library [18]. The server was written in Node JS. The data table is stored in memory as a javascript object, which natively implements a hash table, to permit a short response time when multiple users explore the web application.

The full code to generate the results presented here, obtain statistics and run the web application are freely accessible on Github at https://github.com/arnaudmgh/synergy-screen.

## Additional file

Additional file 1: Table of Synergy Scores. Table of absolute synergy scores and specificity scores for the 5778 combinations tested. **Table S1.** is generated in the source code script combos_script.R with the name combo_ranking_n.syn_score3.csv. (CSV 442 kb)
